# Bidirectional kidney–brain crosstalk in AKI, CKD, and acute brain injury: mechanisms, biomarkers, and translational perspectives

**DOI:** 10.3389/fmed.2026.1995673

**Published:** 2026-10-28

**Authors:** Xiaowei Bao, Rong Jin, Liping Zhang, Linlin Pan, Fengfeng Jiang

**Affiliations:** Department of Neurosurgery, Affiliated Jinhua Hospital, Zhejiang University School of Medicine, Jinhua, Zhejiang, China

**Keywords:** acute brain injury, acute kidney injury, biomarkers, blood-brain barrier, chronic kidney disease, kidney–brain axis, multi-omics, neuroinflammation

## Abstract

The kidney and brain share vascular, metabolic, immune, and neuroendocrine pathways through which dysfunction in either organ can affect the other. Acute kidney injury is associated with delirium and encephalopathy, whereas chronic kidney disease contributes to a longer-term burden of cognitive and cerebrovascular impairment. Retained solutes, drug accumulation, endothelial injury, and systemic inflammation can disrupt the blood-brain barrier and alter glial and neuronal function. Recent experimental studies identify microglial potassium efflux, inflammasome activation, gasdermin D signaling, and ferroptotic stress as components of these responses. Changes in Klotho-FGF23 signaling and impaired perivascular brain clearance provide additional links between renal dysfunction and neurological vulnerability. In the reverse direction, kidney injury after acute brain injury reflects overlapping brain-driven responses, secondary systemic or treatment-related insults, and pre-existing renal and vascular susceptibility. This narrative review brings these mechanisms together with clinical observations and findings from proteomics, metabolomics, and single-cell transcriptomics. It also examines how kidney function affects circulating neurological biomarkers and how study design influences their interpretation. Established management focuses on reversible metabolic disturbances, renal and cerebral perfusion, and medication-related risk. Experimental interventions are discussed according to the disease models and outcomes studied, with emphasis on the evidence needed to translate molecular targets into improvements in cognition, neurological recovery, and kidney function.

## 1 Introduction

The kidney and brain maintain homeostasis through connected vascular, metabolic, immune, and neuroendocrine pathways. Dysfunction in one organ can disturb the function of the other. This relationship is evident across acute kidney injury (AKI), chronic kidney disease (CKD), stroke, intracerebral hemorrhage, subarachnoid hemorrhage, and traumatic brain injury (1–6).

Kidney dysfunction is associated with acute delirium and encephalopathy during AKI and with cognitive impairment and cerebrovascular disease during CKD (7–10). Conversely, AKI complicates acute brain injury and is associated with poorer survival and neurological recovery (2, 11–13). Uremic solutes, metabolic disturbances, systemic inflammation, endothelial injury, autonomic activation, and treatment-related stress connect these clinical phenotypes, but their relative contributions differ between acute and chronic disease.

This review examines both directions of kidney-brain crosstalk, linking clinical phenotypes and biomarkers to experimental mechanisms. It focuses on inflammatory and metabolic signaling, regulated cell death, endocrine changes, and emerging evidence of impaired cerebral clearance. Multi-omics findings are considered alongside the study designs that support them. The therapeutic discussion separates established management from interventions being developed in experimental models.

## 2 Literature search strategy

The initial literature search covered PubMed, Web of Science, and Google Scholar for English-language publications from database inception to June 2026. Targeted searches of PubMed and publisher websites were updated through 6 October 2026 during revision. Search terms combined kidney or brain conditions (“acute kidney injury”, “chronic kidney disease”, “stroke”, “intracerebral hemorrhage”, and “traumatic brain injury”) with “kidney-brain crosstalk”, “blood-brain barrier”, “uremic toxins”, “microglia”, “NLRP3”, “gasdermin D”, “ferroptosis”, “Klotho”, “FGF23”, “glymphatic”, “proteomics”, “metabolomics”, or “single-cell RNA sequencing”. Clinical studies, mechanistic animal and cell studies, relevant evidence syntheses, and clinical guidance were selected for their contribution to the pathways discussed. Reference lists were screened for additional studies. Sources were interpreted in their original disease and experimental context; citations that did not support the associated claim were corrected or excluded. Findings were synthesized by disease context, study design, and outcome; no pooled effect estimate or formal risk-of-bias assessment was undertaken.

## 3 Clinical context and biomarker landscape of kidney–brain crosstalk

### 3.1 Bidirectional disease burden

Kidney-to-brain effects differ with the tempo and severity of renal dysfunction. AKI is associated with delirium, impaired attention, altered consciousness, and severe uremic encephalopathy (7, 8). CKD is associated with deficits in executive function, attention, and memory, alongside an increased burden of cerebrovascular disease (9, 10, 14–17). Age, diabetes, hypertension, anemia, medications, and critical illness modify these phenotypes and should be considered when interpreting neurological symptoms.

The association can extend beyond the acute episode: a longitudinal cohort linked AKI with subsequent stroke risk (18). This observation places acute renal injury within a longer-term vascular trajectory, without identifying a single renal mediator.

AKI occurs after ischemic and hemorrhagic stroke, subarachnoid hemorrhage, and traumatic brain injury, with incidence varying by case mix and diagnostic criteria (2, 11–13, 19–22). Brain-driven autonomic, endocrine, and inflammatory responses coexist with hypotension, infection, procedures, and medication exposure. Pre-existing CKD and vascular disease increase susceptibility to both cerebral and renal injury. These overlapping explanations, developed in Section 5, are important when interpreting associations between AKI and neurological outcome.

Traumatic brain injury cohorts have also associated the triglyceride-glucose index and serum magnesium with AKI risk (23, 24). These are clinical risk-marker associations rather than evidence that either marker transmits injury from brain to kidney.

### 3.2 Biomarkers and interpretive caveats

Serum creatinine, blood urea nitrogen, estimated glomerular filtration rate (eGFR), urine output, electrolytes, and plasma osmolality describe renal clearance and homeostatic control, rather than neurological injury itself (1, 2, 7). Albuminuria is associated with cerebral small vessel disease and cognitive impairment and can indicate a shared microvascular substrate (14, 15). Inflammatory proteins and endothelial junction markers provide complementary mechanistic information. For example, experimental urea exposure has been linked to matrix metalloproteinase-2 (MMP-2) activity, reduced claudin-5 and platelet endothelial cell adhesion molecule-1, and BBB leakage (25). These readouts require validation against clinical outcomes before use in routine risk prediction.

Blood neurofilament light chain and phosphorylated tau concentrations increase as kidney function declines. Human studies show that renal function influences these measurements, so elevated concentrations should not be interpreted as evidence of increased brain pathology without considering eGFR and other clinical data (26, 27). KIM-1 was originally characterized as an injury-responsive renal epithelial protein (28). Olink profiling subsequently identified elevated plasma KIM-1 in an intracerebral hemorrhage cohort despite similar conventional renal indices (29). This finding is a candidate signal for validation, not proof of a brain-specific source. Table 1 summarizes representative mechanisms and readouts; Figure 1 outlines the relationship between renal dysfunction, circulating signals, and BBB injury.

**Table 1:** Mechanisms, translational readouts, and interpretive constraints in kidney–brain crosstalk.

Context	Mechanism	Representative readouts	Evidence and interpretation
AKI to brain	Solute and drug retention; inflammation; barrier injury	Renal indices, electrolytes, inflammatory proteins	Clinical delirium association; direct cerebral effects in renal ischemia models (8, 39).
CKD to brain	Microvascular disease; toxin-endothelial and glial signaling	Albuminuria, BBB markers, NfL and p-tau	Human vascular associations and experimental mechanisms; blood neurological markers depend on kidney function (14, 26, 27, 53).
Brain injury to kidney	Autonomic and inflammatory responses; secondary insults; baseline susceptibility	Perfusion, urine output, creatinine, tubular markers	Plasma-transfer evidence supports tubular injury; clinical AKI remains multifactorial (2, 101).
Gut-derived signaling	Microbial metabolites and reduced renal clearance	Indoxyl sulfate, phenylacetylglutamine, TMAO	Experimental toxin effects and pediatric metabolite-cognition associations (48, 91).
Klotho-FGF23	Endocrine and tissue-specific Klotho changes	Renal/cortical Klotho, FGF23, phosphate	CKD animal findings; population-dependent human FGF23 associations (93, 95, 96).
Cerebral clearance	Perivascular fluid exchange	DTI-ALPS, cognition and imaging	Small human cohorts; an indirect diffusion measure, not direct toxin clearance (98–100).
Cell-resolved omics	Tubular injury states; glial-neuronal responses; synaptic engulfment	Sox9/Vcam1 states; potassium signaling; Ccl4/Ccr5	Renal and brain injury studies plus normal-tissue computational predictions (53, 69, 137, 138).

AKI, acute kidney injury; BBB, blood-brain barrier; CKD, chronic kidney disease; Ccl4, C-C motif chemokine ligand 4; Ccr5, C-C chemokine receptor 5; DTI-ALPS, diffusion tensor imaging analysis along the perivascular space; eGFR, estimated glomerular filtration rate; FGF23, fibroblast growth factor 23; KIM-1, kidney injury molecule-1; NfL, neurofilament light chain; TMAO, trimethylamine N-oxide.

**Figure 1:**
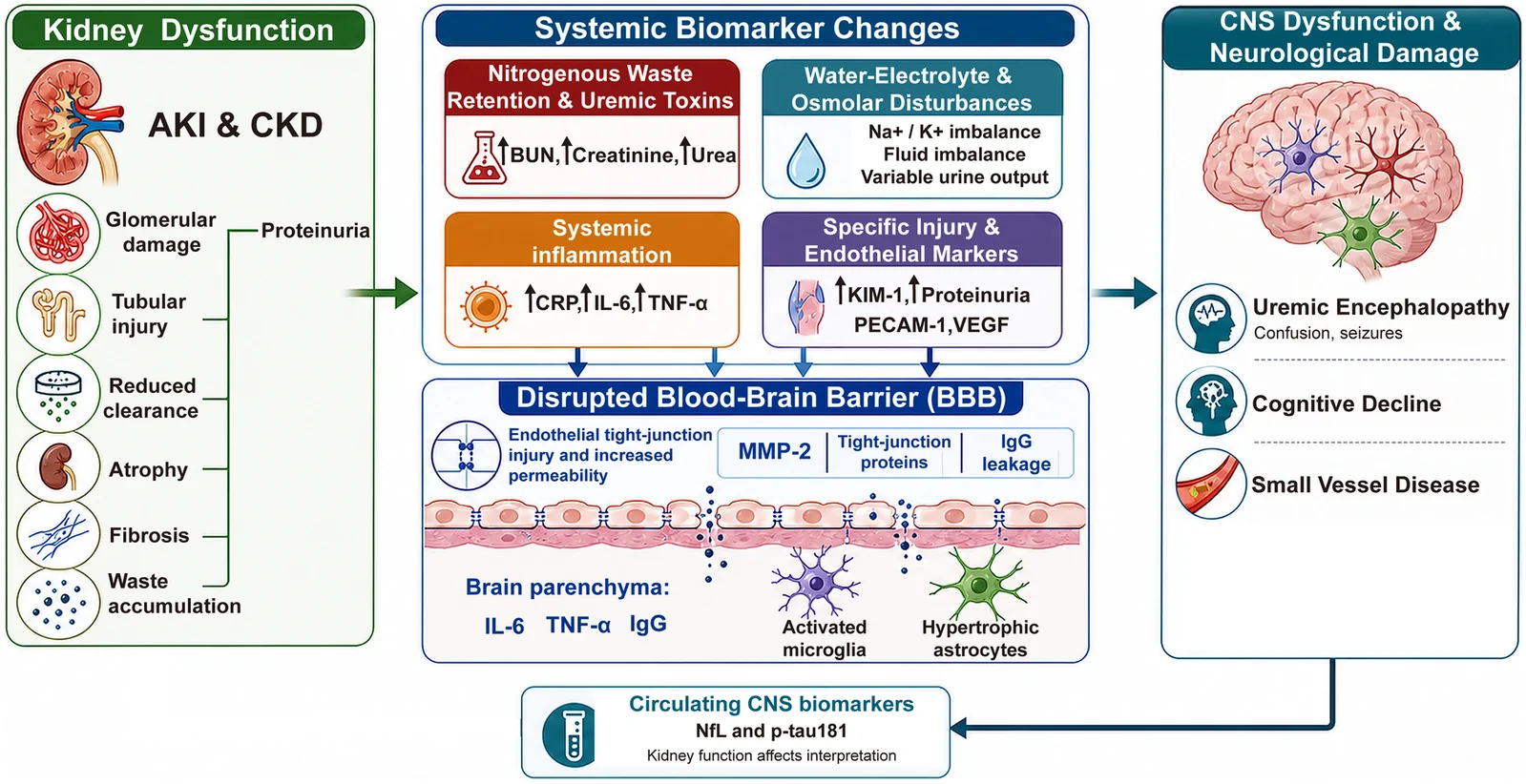
Circulating biomarkers and blood-brain barrier changes associated with kidney dysfunction. AKI and CKD alter solute handling, inflammatory signaling, and fluid-electrolyte balance. Endothelial and glial responses can connect these disturbances to neurological dysfunction. The schematic combines clinical phenotypes with experimental pathways; individual biomarkers are not interchangeable measures of causal inter-organ signaling. Sodium, potassium, and urine-output changes vary with disease context. Circulating NfL and p-tau181 require interpretation alongside kidney function and clinical findings. The CNS-biomarker panel does not imply that these proteins cause kidney injury. AKI, acute kidney injury; BBB, blood-brain barrier; BUN, blood urea nitrogen; CKD, chronic kidney disease; CNS, central nervous system; CRP, C-reactive protein; IgG, immunoglobulin G; IL-6, interleukin-6; KIM-1, kidney injury molecule-1; MMP-2, matrix metalloproteinase-2; NfL, neurofilament light chain; PECAM-1, platelet endothelial cell adhesion molecule-1; p-tau181, tau phosphorylated at threonine 181; TNF-α, tumor necrosis factor-α; VEGF, vascular endothelial growth factor.

The biological range of KIM-1 further limits organ-specific interpretation. It has been studied as a tissue and urinary marker in renal cell carcinoma, as a predictor of graft loss in transplant recipients, and as a mediator of tubular fatty-acid uptake in diabetic kidney disease (30–32). Genetic and circulating biomarker frameworks for cognitive dysfunction in CKD therefore require validation against both renal pathology and neurological outcomes (33).

## 4 Kidney-to-brain crosstalk: acute and chronic mechanisms

### 4.1 AKI-associated acute brain dysfunction

AKI is independently associated with delirium and coma in critically ill patients, and the association strengthens with AKI severity (8). Sepsis, shock, sedation, and mechanical ventilation commonly coexist, making the renal contribution difficult to isolate (7, 34–36). A new change in attention or arousal should prompt evaluation of uremia, retained drugs, electrolytes, acid-base status, infection, oxygenation, and structural neurological disease. Neuroinflammatory models of delirium provide a framework for these interacting insults rather than a kidney-specific explanation (37).

Rapid changes in the internal milieu contribute to the acute phenotype. Reduced renal elimination increases exposure to uremic solutes and renally cleared sedatives, opioid metabolites, and antimicrobial agents (7, 38). Renal ischemia models also demonstrate cerebral cytokine responses, glial activation, BBB permeability changes, and altered behavior (39, 40). These processes interact with changes in sodium, osmolality, and acid-base balance. Figure 2 summarizes the resulting acute immune-metabolic response.

**Figure 2:**
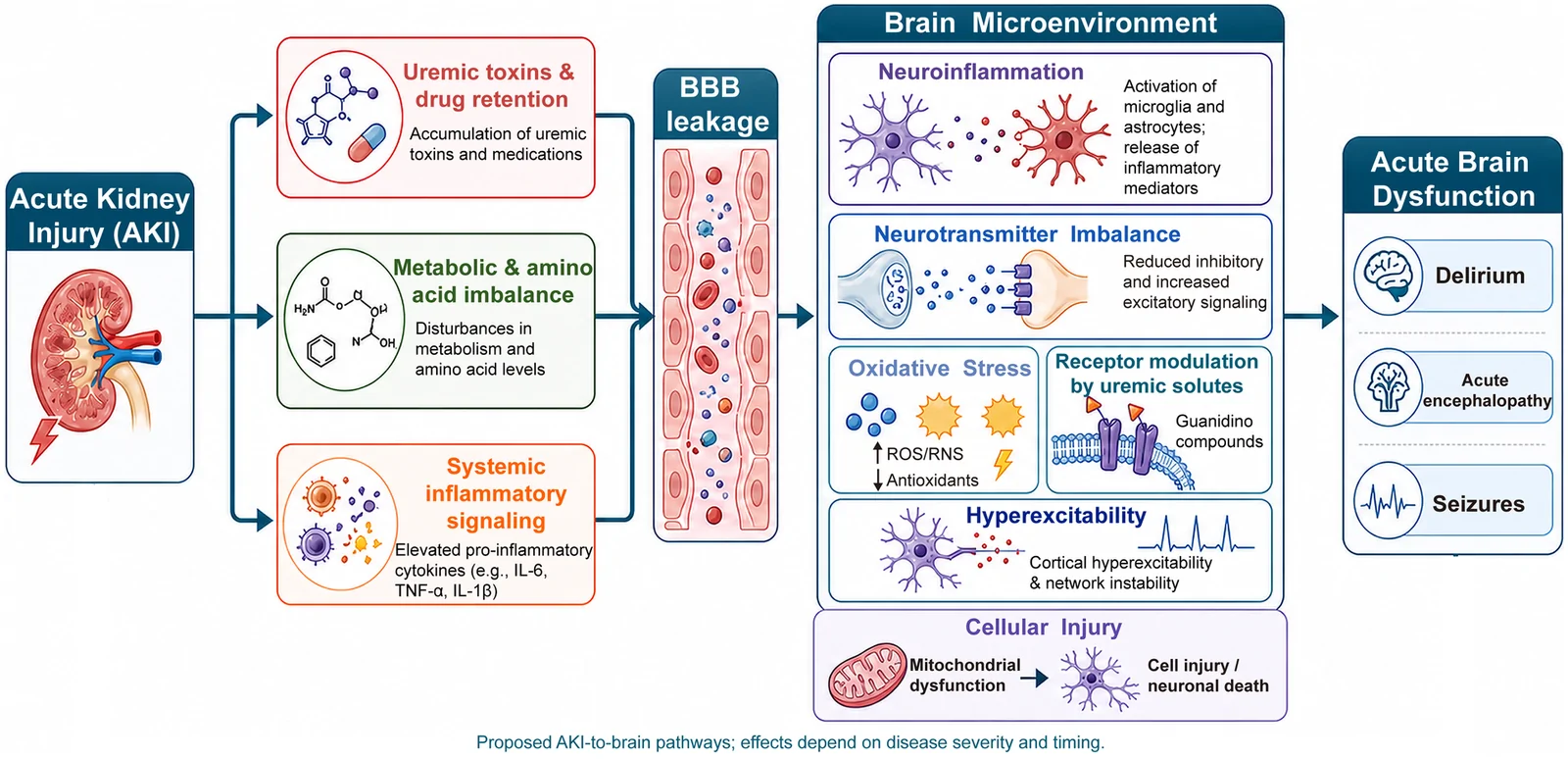
Proposed pathways connecting acute kidney injury to acute brain dysfunction. Reduced renal elimination, metabolic disturbances, and systemic inflammatory signaling can affect the brain through endothelial, glial, and neuronal responses. Experimental findings support altered excitatory and inhibitory receptor signaling, oxidative stress, and mitochondrial injury; these responses depend on the model, timing, and severity of disease. Guanidino compounds can modulate receptor signaling, rather than producing uniform blockade of all neurotransmitter receptors. Delirium, encephalopathy, and seizures are clinical manifestations that also require assessment of coexisting systemic and neurological causes. The schematic depicts AKI-to-brain pathways and does not establish a causal feedback loop from these manifestations to the kidney. AKI, acute kidney injury; BBB, blood-brain barrier; IL-1β, interleukin-1β; IL-6, interleukin-6; RNS, reactive nitrogen species; ROS, reactive oxygen species; TNF-α, tumor necrosis factor-α.

### 4.2 Retained solutes and damage-associated inflammatory signaling

Retained solutes affect the brain through different mechanisms. Guanidino compounds alter neuronal receptor activity, whereas indoxyl sulfate and p-cresyl sulfate connect impaired clearance to endothelial and inflammatory responses (38, 41, 42). Drug accumulation adds a clinically important, potentially reversible source of neurotoxicity (7). The speed of accumulation helps distinguish AKI-associated encephalopathy from the prolonged vascular and metabolic exposures of CKD.

Exposure depends on the concentration and biological properties of each retained solute. Published comparisons of normal and uremic concentrations help define physiologically relevant experimental doses (43). Advanced glycation end products add a receptor-mediated inflammatory and oxidative component to chronic uremic toxicity (44). In a clinical CKD study, indoxyl sulfate and oxidative-stress measures were examined in relation to peripheral nerve dysfunction (45); peripheral neuropathy should be distinguished from a demonstrated cerebral effect.

Kidney injury can generate inflammatory signals as well as impair their elimination (46). Renal ischemia in mice induces systemic inflammatory transcriptional responses and cerebral inflammation (39, 47). In experimental CKD, urea exposure increases MMP-2 activity and reduces endothelial junction proteins, providing a specific route to BBB disruption (25). Indoxyl sulfate offers another route: experimental renal dysfunction and toxin exposure impair the BBB and cognition through aryl hydrocarbon receptor (AhR) signaling, with protection in AhR-deficient mice (48). Solute-mediated and inflammatory mechanisms therefore converge at the cerebral endothelium.

The systemic response is not confined to the brain. Renal ischemia-reperfusion amplified antigen-specific humoral responses in mice (49), while renal ischemia or nephrectomy caused cytokine-dependent intestinal and hepatic injury (50). Separate renal ischemia studies demonstrated macrophage-dependent pulmonary permeability changes and a lung transcriptional response that differed from bilateral nephrectomy (51, 52). These comparisons support a distinction between injury-generated inflammatory signals and loss of clearance; they do not establish that identical pathways operate in every distant organ.

Microglial responses link renal dysfunction to neuronal dysfunction. In a CKD mouse study, KCa3.1-dependent potassium efflux promoted microglial IL-1β signaling and altered neuronal potassium handling. Neuron-specific IL-1 receptor deletion and pharmacological interventions improved cognition, supporting a functional microglia-neuron pathway (53–55).

A follow-up CKD study localized IL-1β maturation to a cathepsin C-caspase-8 pathway in microglia. Microglia-specific caspase-1 deletion did not improve cognition, whereas cathepsin C or caspase-8 depletion blocked IL-1β maturation (56). Thus, brain NLRP3 activation and microglial cytokine processing represent distinct, cell-dependent components of the response.

A complementary study used repeated peripheral lipopolysaccharide exposure rather than renal injury. Microglial NLRP3-gasdermin D (GSDMD) signaling increased GDF-15, perivascular CXCL chemokines, and metalloproteinases, recruiting CXCR2-expressing neutrophils and disrupting the BBB. NLRP3 or microglial GSDMD deficiency reduced barrier injury; notably, the pathway did not require IL-1β (57). This is a testable mechanism for inflammation-associated kidney-to-brain signaling, although it has not yet been demonstrated directly in a renal-injury model. GSDMD is a pyroptotic pore-forming effector, but its activation alone should not be equated with completed microglial lysis.

Barrier responses also depend on the inflammatory stimulus. Systemic LPS, IL-1β, TNF-α, and IL-6 produced different peripheral and central inflammatory profiles in mice (58). TNF-α contributed to BBB permeability in bacterial sepsis (59), whereas a viral model identified a perforin-dependent CD8T-cell route (60). Hyperosmolar mannitol experiments implicated VEGF in barrier disruption (61). These nonrenal models provide mechanistic comparisons rather than direct evidence of renal-to-brain causation.

### 4.3 Oxidative stress, mitochondrial injury, and regulated cell death

Mitochondrial injury can sustain oxidative stress and inflammatory signaling (62, 63). In ischemic AKI, mitochondrial reactive oxygen species disrupt TFAM-dependent mitochondrial DNA maintenance (64). A CKD mouse model also links hypercalcemia to Drp1-dependent mitochondrial fragmentation and neuronal injury (65). These examples connect specific metabolic disturbances to cellular injury rather than treating oxidative stress as a single uniform pathway.

Ferroptosis is regulated cell death caused by iron-dependent accumulation of lipid peroxides. It differs from caspase-mediated apoptosis and becomes prominent when glutathione-dependent antioxidant defenses, including glutathione peroxidase 4 (GPX4), fail (66, 67). Gpx4 inactivation causes acute tubular injury in mice, and ferroptosis contributes to synchronized tubular death during renal ischemia-reperfusion (67, 68). Persistent ferroptotic stress can also maintain pro-inflammatory proximal tubular states during maladaptive repair, connecting acute injury to chronic inflammation and fibrosis (69, 70).

Evidence in the brain comes from separate disease models. Ferroptosis inhibition reduces experimental ischemic stroke injury, while forebrain neuronal Gpx4 deletion produces progressive neurodegeneration and cognitive impairment (71, 72). Together with renal studies, these findings identify a shared vulnerability to lipid peroxidation. Establishing ferroptosis as an organ-to-organ mediator requires renal-injury models in which cell-specific perturbations also alter neurological outcomes.

Oxidative barrier injury can also arise independently within the brain. A humanized NOX5 mouse model and human barrier experiments linked calcium-dependent NOX5 activity to postreperfusion BBB leakage in stroke (73). This complements the mitochondrial evidence by identifying a separate ROS-generating pathway at the neurovascular interface.

### 4.4 Chronic CKD, neurovascular injury, and gut-derived immune signaling

CKD-related neurological impairment develops against a background of vascular disease, retained solutes, inflammation, and treatment-associated circulatory stress. Clinical cohorts report cognitive deficits as kidney function declines, but the affected domains and trajectories vary (9, 10, 74). Compared with the rapidly changing neurological manifestations of AKI, chronic exposures accumulate over months or years and interact with aging, diabetes, hypertension, and anemia. Diabetes-related cerebral microvascular disease is an important overlapping contributor (75).

The vascular and solute-related components can be distinguished by their supporting evidence. Associations of albuminuria with white-matter lesions, lacunes, and cognitive impairment support shared renal and cerebral microvascular pathology; they do not identify a particular circulating neurotoxin (14, 15, 76–78). Human hemodialysis studies document intradialytic decreases in cerebral blood flow and cerebral oxygenation, with substantial variation in the lower limit of cerebral autoregulation (79, 80). Impaired cerebral autoregulation and repeated perfusion stress can therefore expose susceptible brain tissue to ischemic injury.

Inflammatory and lipid-related vascular susceptibility is supported by additional clinical evidence. IL-6 concentrations were associated with cardiovascular events across CKD strata in a chronic coronary syndrome cohort (81), and remnant cholesterol was associated with CKD in an analysis that examined inflammatory mediation (82). These studies characterize systemic risk rather than a brain-specific biomarker.

Solute-mediated mechanisms have stronger support from experimental perturbation. Indoxyl sulfate activates AhR-dependent BBB injury and can increase endothelial E-selectin and leukocyte-endothelial interactions (48, 83). Thus, vascular and toxin-mediated effects are not mutually exclusive: retained metabolites can themselves injure the neurovascular interface. The distinction is between evidence for shared susceptibility in clinical populations and evidence for a specific molecular effect in an experimental model.

Neurotransmitter changes are also mechanism- and time-dependent. Experimental studies of uremic guanidino compounds describe NMDA receptor activation and inhibition of GABA-A receptor signaling, shifting networks toward excitation rather than demonstrating a uniform rise in glutamate or fall in GABA concentrations (41, 84). In rats examined 48 h after renal ischemia, dopamine turnover decreased in the striatum, mesencephalon, and hypothalamus, whereas noradrenaline and serotonin turnover remained unchanged (85). This is an acute renal-failure finding; it should not be generalized to all chronic uremic neurotransmitter systems.

Other rat experiments identified time- and region-dependent neuronal activation after nephrectomy, ureteral ligation, or toxic renal injury (86). An acute-vs.-chronic uremia study reported elevated plasma catecholamines alongside depletion of brain catecholamines (87). Concentration, turnover, and neuronal activation are different readouts, which helps explain apparently different directions across models. At the BBB, cationic amino-acid transport through system y+ provides a further example of selective substrate access rather than unrestricted diffusion (88).

The gut supplies an additional source of retained metabolites. Altered microbial metabolism and reduced renal clearance increase exposure to indole-, phenol-, and trimethylamine-derived compounds (89, 90). These metabolites can influence endothelial activation and inflammatory signaling, as illustrated by indoxyl sulfate, while human metabolomic studies link selected gut-derived molecules to neurological phenotypes (83, 91). Figure 3 summarizes how these exposures intersect with chronic neurovascular and glial injury.

**Figure 3:**
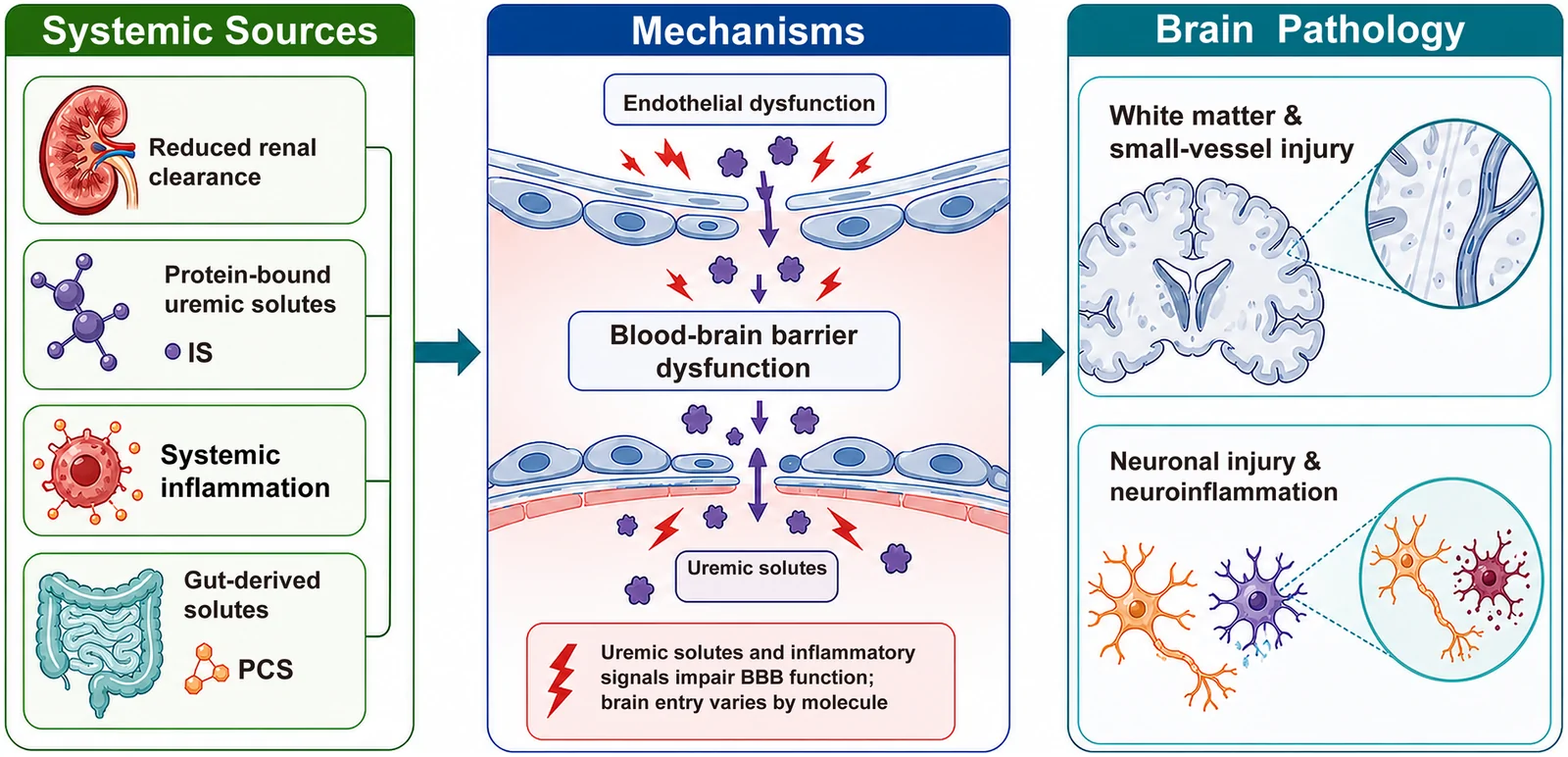
Uremic solutes, endothelial dysfunction, and cerebral injury in chronic kidney disease. Reduced renal clearance and gut-derived solute production contribute to retention of protein-bound uremic solutes, including IS and PCS. Uremic and inflammatory signals can impair endothelial function and BBB integrity, with potential consequences for small vessels, white matter, and neuronal-glial homeostasis. Brain entry and effects vary among molecules; the schematic does not imply that all circulating solutes or cytokines traverse the BBB in the same manner. The pathways integrate experimental mechanisms with clinical associations rather than establish a single obligatory sequence. BBB, blood-brain barrier; IS, indoxyl sulfate; PCS, p-cresyl sulfate.

### 4.5 Klotho-FGF23 signaling and cerebral clearance

Endocrine changes provide another connection between CKD and brain vulnerability. Membrane-bound *α*-Klotho acts as a co-receptor that enables canonical fibroblast growth factor 23 (FGF23) signaling (92). In adenine-induced CKD, rats showed cognitive impairment together with reduced renal and cortical membrane-bound Klotho and increased circulating FGF23 (93). The cortical finding concerns locally measured Klotho; it does not establish transfer of renal Klotho into the brain or identify which endocrine change causes the cognitive phenotype.

Klotho-associated cognition also has a synaptic component. Systemic Klotho overexpression in mice enriched synaptic GluN2B, an NMDA receptor subunit, and enhanced hippocampal long-term potentiation and learning. GluN2B blockade abolished these effects (94). This non-CKD experiment identifies a neural functional mechanism alongside Klotho's endocrine role; its contribution to cognitive impairment associated with reduced cortical Klotho in CKD requires direct testing (93).

Human FGF23 findings depend on the population studied. Higher FGF23 was associated with poorer memory in a hemodialysis cohort, whereas a larger cohort of older adults found no adjusted association with incident cognitive impairment (95, 96). FGF23 and Klotho should therefore be evaluated alongside phosphate status, renal function, vascular disease, and the cognitive domain assessed, rather than treated as interchangeable markers of neurotoxicity.

Cerebral solute clearance may also be affected. Experimental work describes aquaporin-4-associated exchange between cerebrospinal and interstitial fluid along perivascular pathways (97). Studies in advanced kidney disease report lower diffusion tensor imaging analysis along the perivascular space (DTI-ALPS) indices, including lower indices in CKD patients with cognitive impairment (98, 99). A small longitudinal study found no improvement three months after dialysis initiation (100). DTI-ALPS measures directional water diffusion and is an indirect proxy, influenced by tissue and vascular properties. These findings support investigation of impaired brain clearance in CKD, but do not directly measure toxin removal or establish a causal Klotho-glymphatic pathway.

## 5 Brain-to-kidney crosstalk after acute brain injury

AKI following acute brain injury can be understood through three interconnected frameworks. First, the cerebral lesion can initiate sympathetic, neuroendocrine, and circulating inflammatory responses that affect the kidney. Experimental exposure of renal tubular cells to plasma from severe traumatic brain injury patients provides evidence of a circulating injurious signal, although it does not isolate a single brain-derived mediator (101).

Second, AKI can arise indirectly from shock, bleeding, surgery, sepsis, ventilation, or treatment-related changes in perfusion and osmolality (2, 19). Third, CKD, hypertension, diabetes, aging, and pre-existing vascular injury confer shared baseline vulnerability to both cerebral events and AKI (2, 14). The balance between these explanations differs among patients. Longitudinal analyses that account for baseline renal function, lesion severity, systemic instability, and treatment exposure are needed to estimate the additional contribution of the cerebral injury itself. Obesity-related adipose inflammation and insulin resistance provide additional context for shared metabolic and vascular vulnerability (102). Figure 4 summarizes the principal pathways and modifying factors linking acute brain injury to AKI.

**Figure 4:**
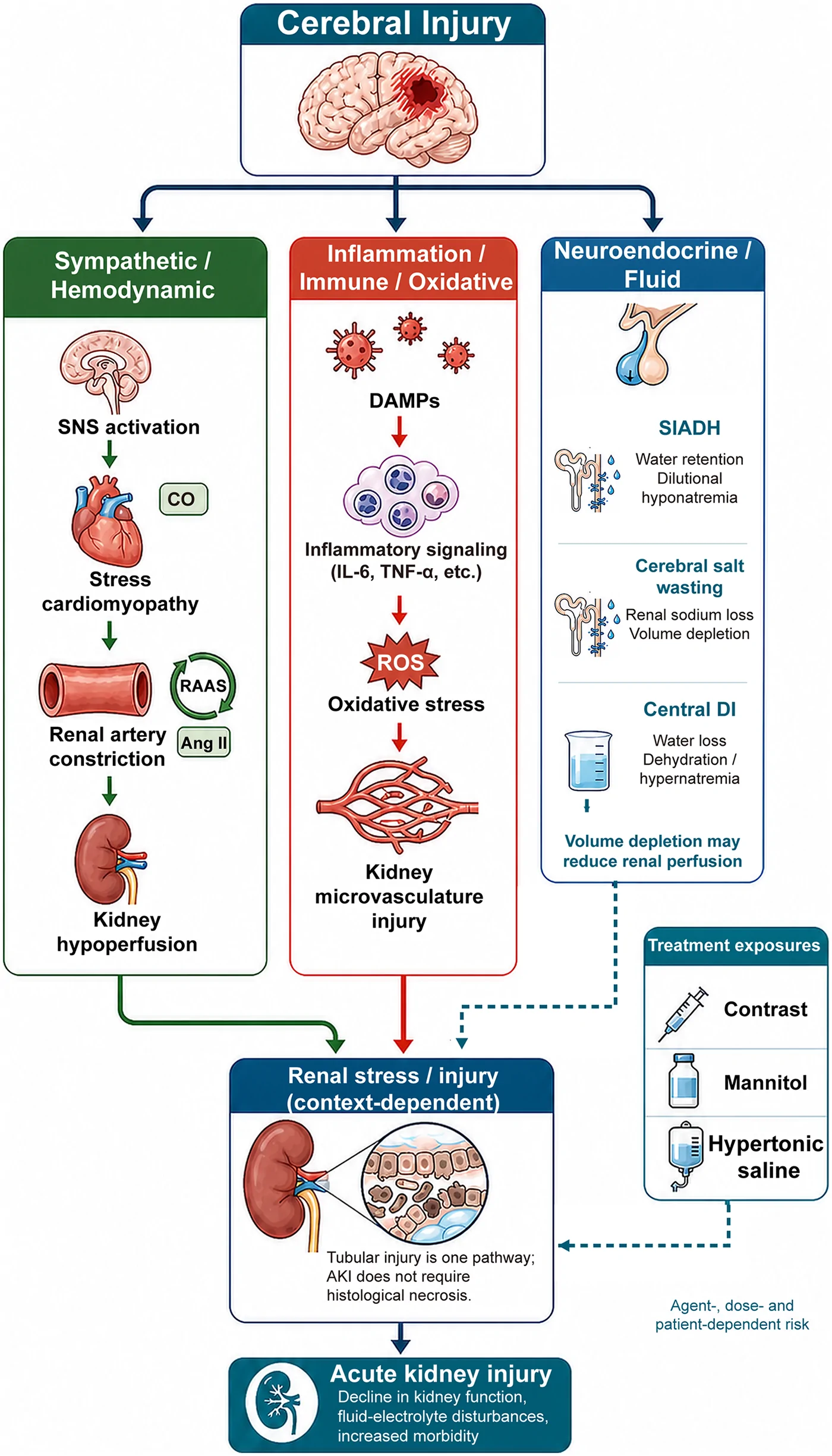
Pathways and modifying factors in AKI after acute brain injury. Sympathetic and circulatory changes, systemic inflammation, endocrine disturbances, and treatment exposures can affect renal function after acute brain injury. SIADH causes water retention and dilutional hyponatremia, whereas cerebral salt wasting and central DI can cause volume depletion. The endocrine branch therefore distinguishes electrolyte imbalance from conditions that may reduce renal perfusion. Dashed connections indicate context-dependent contributions of volume depletion and treatment exposures to renal stress or injury. Iodinated contrast, mannitol, and hypertonic saline have distinct renal risk profiles and are not depicted as uniformly nephrotoxic. AKI is a clinical syndrome that does not require histologically demonstrated tubular necrosis. AKI, acute kidney injury; Ang II, angiotensin II; CO, cardiac output; DAMPs, damage-associated molecular patterns; DI, diabetes insipidus; IL-6, interleukin-6; RAAS, renin-angiotensin-aldosterone system; ROS, reactive oxygen species; SIADH, syndrome of inappropriate antidiuresis; SNS, sympathetic nervous system; TNF-α, tumor necrosis factor-α.

### 5.1 Sympathetic and hemodynamic stress

Sympathetic activation after acute cerebral injury increases catecholamine signaling and can alter cardiac output and vascular resistance (2, 3, 19). Stress cardiomyopathy has been documented after intracerebral hemorrhage (103). Renal sympathetic *β*1-adrenergic signaling and reduced renal perfusion stimulate renin release, providing a physiological route to renin-angiotensin-aldosterone system (RAAS) activation (3). The contribution of this route to brain-injury-associated AKI varies with blood pressure, cardiac function, volume status, and treatment; it is not established as the dominant mechanism in every patient.

Experimental RAAS findings vary with the model. Angiotensin-1-7 reduced the creatinine rise after traumatic brain injury in rats, although the study did not identify corresponding overt renal histological damage (104). Endothelial prolyl hydroxylase-2 deletion attenuated angiotensin-II-associated renal injury in mice (105), and renal denervation reduced aldosterone-related changes in an angiotensin-II hypertension model (106). These experiments identify modifiable pathways, not a uniform endocrine sequence after human brain injury.

At the bedside, impaired forward flow, renal vasoconstriction, and venous congestion can all reduce effective renal perfusion (2, 19). Blood-pressure treatment must therefore balance cerebral goals with renal tolerance. An intracerebral hemorrhage cohort associated larger acute systolic pressure reductions with AKI, illustrating an indirect, treatment-associated perfusion pathway (107).

Cardiac complications after subarachnoid hemorrhage and cognitive dysfunction in heart failure illustrate a possible cardiac intermediate within the broader organ network (108–110). Cardiac-surgery studies link AKI to perioperative stress, reduced catecholamine degradation, and elevated central venous pressure (111–113). These are adjacent clinical contexts that help identify confounders, not direct estimates of brain-injury-associated renal risk.

Catecholamine handling also has potentially opposing effects. In rats with renal ischemia-reperfusion, the monoamine oxidase inhibitor isatin reduced superoxide production but increased norepinephrine overflow and worsened renal injury (114). A reduction in an oxidative readout therefore does not necessarily predict renal protection when hemodynamic signaling changes simultaneously.

### 5.2 Neuroendocrine and fluid-electrolyte dysregulation

Hypothalamic-pituitary injury can disturb arginine vasopressin (AVP) and cortisol regulation (115, 116). Syndrome of inappropriate antidiuresis (SIADH) causes water retention and dilutional hyponatremia, usually without volume depletion. Cerebral salt wasting instead involves renal sodium loss and extracellular volume contraction, which can reduce renal perfusion. Central diabetes insipidus produces dilute polyuria, with dehydration and hypernatremia when water replacement is inadequate (19, 116).

SIADH and cerebral salt wasting can both present with hyponatremia, concentrated urine, and elevated urine sodium despite requiring different fluid strategies. Cortisol deficiency can also resemble SIADH. Serial assessment of fluid balance, volume status, urine output, serum and urine osmolality, sodium, and adrenal function helps distinguish these conditions (116). Hyperosmolar treatment introduces separate changes: mannitol promotes osmotic diuresis, while hypertonic saline raises sodium and chloride concentrations. Their kidney-related risks are considered separately below (117).

### 5.3 Systemic inflammation, immune dysfunction, and renal susceptibility

Circulating inflammatory signals can change the renal microvascular and tubular response. Cytokine-induced endothelial activation promotes leukocyte adhesion, while leukocyte oxidants and proteases can amplify epithelial injury; inflammatory signaling can also disrupt tubular transport and cellular survival (118). In a study of severe traumatic brain injury, plasma exposure increased neutrophil adhesion to human proximal tubular cells, reduced epithelial electrical resistance and megalin expression, and induced apoptosis (101). This links patient-derived circulating factors to tubular dysfunction, but does not establish that all measured factors originated in the injured brain.

Renal experiments identify specific effectors within this response. Dipeptidase-1 deficiency or inhibition reduced leukocyte recruitment and renal ischemic injury (119), while macrophage KLF4 limited TNF-dependent renal injury and fibrosis (120). Procalcitonin-related vascular barrier experiments provide a further systemic endothelial mechanism (121). None of these findings alone identifies the initiating cerebral signal.

Damage-associated signals offer additional routes to inflammatory amplification. Mitochondrial molecules and extracellular HMGB1 can activate innate immune responses (122, 123). In radiation-induced brain injury, extracellular ATP-P2X7 signaling promoted microglial activation and neuronal damage (124). The radiation model demonstrates a local damage-signaling pathway; whether the same mediators reach and injure the kidney after acute brain injury requires separate testing.

Severe central nervous system injury can subsequently suppress peripheral immune function, increasing susceptibility to infection (125, 126). Sepsis then adds inflammatory, microcirculatory, and hemodynamic routes to AKI. This sequence links a brain-driven immune response to an indirect renal insult rather than attributing all renal injury to immediate cytokine spillover.

Hyperosmolar agents have different renal risk profiles. Mannitol can cause osmotic diuresis and volume depletion; impaired renal elimination can increase osmotic exposure and tubular stress. Hypertonic saline instead increases sodium and chloride, with severe hypernatremia and hyperchloremia associated with AKI in neurocritical care guidance (117, 127). Baseline CKD, cumulative dosing, concurrent diuretics, and fluid balance modify these risks. Monitoring should match the agent and the patient's perfusion and intracranial-pressure goals.

Iodinated contrast is a separate diagnostic exposure. Contrast-associated AKI describes a temporal association, whereas contrast-induced AKI implies causation. The renal risk of modern intravenous contrast has often been overestimated because acute illness itself causes AKI (128). Active AKI, severe CKD, hypovolemia, and other nephrotoxic exposures warrant individualized assessment. Hyperosmolar treatment, contrast administration, and systemic instability may occur together, but their contributions should be evaluated separately rather than grouped under a single nephrotoxic mechanism.

A retrospective emergency-department stroke-protocol cohort found that post-contrast AKI differed by baseline kidney function, with greater risk in the more advanced CKD group (129). Because all patients underwent contrast-enhanced imaging, the study informs risk stratification but does not isolate the causal contribution of contrast from the acute illness.

## 6 Multi-omics insights into kidney–brain crosstalk

Multi-omics can connect circulating signals with cellular states and clinical phenotypes. Its value lies in resolving mechanisms and generating biomarker hypotheses that can be tested in longitudinal cohorts (130, 131). Interpretation depends on whether measurements come from a renal-injury model, a neurological cohort, or computational integration of separate datasets.

### 6.1 Proteomics and inflammatory biomarker panels

Proteomic studies identify proteins associated with renal injury, inflammation, and neurological disease. The Olink intracerebral hemorrhage study identified plasma KIM-1 despite similar conventional renal indices, alongside IL-6, pentraxin 3, and galectin-9 in a combined circulating panel (29). These proteins are candidates for validation against renal function, illness severity, and neurological outcomes.

Matsuki et al. used differential extraction and mass spectrometry to characterize soluble and sarkosyl-insoluble hippocampal proteins in adenine-induced CKD mice. Insoluble tau and RNA-binding proteins, including small nuclear ribonucleoprotein U1 subunit 70 (SNRNP70), accumulated, while IgG enrichment supported BBB leakage. The accompanying endothelial experiments linked urea exposure to MMP-2 activity and loss of junction proteins (25). These findings connect a brain proteomic phenotype to a testable renal solute-mediated mechanism.

Tau normally participates in neuronal microtubule regulation, while its pathological modification and aggregation are central to several neurodegenerative processes (132). The CKD proteomic observation should therefore be interpreted as a measured change in protein solubility, not by itself as a diagnosis of a specific tauopathy.

A useful proteomic signature must improve prediction beyond eGFR, albuminuria, illness severity, and neurological assessment. Standardized sampling, independent replication, and prospective evaluation are central to omics-based kidney classification (130, 131). Combining blood and urine profiles with imaging and, when clinically available, cerebrospinal fluid measurements could help distinguish reduced clearance from active tissue injury.

### 6.2 Metabolomics and the gut–kidney–brain axis

Metabolomics captures gut-derived and host metabolites that are not represented by creatinine or urea. Experimental profiling documents microbiota-dependent inter-organ metabolite transport (133). In CKD, phenylacetylglutamine is associated with cardiovascular events and mortality, while TMAO concentrations vary with kidney disease stage (134, 135). These associations establish systemic relevance but do not by themselves demonstrate a neurological effect.

Lee et al. examined metabolomic associations in the CKiD and NiCK pediatric cohorts. Phenylacetylglutamine, indoleacetylglutamine, and TMAO were associated with parent-rated executive function; *γ*-glutamyl amino acids and aconitate were linked to intelligence measures (91). These signals point to microbial metabolism, glutathione-related redox balance, and mitochondrial energy metabolism. The analyses included models with and without eGFR adjustment, and associations differed across samples. They identify candidate pathways, rather than a consistent rule that every retained metabolite worsens cognition or an established causal effect in children.

Diet, antibiotics, medication use, residual renal function, and dialysis influence metabolite concentrations. Future studies should track metabolite and cognitive changes together, test prediction beyond kidney function, and determine whether lowering a candidate metabolite improves neurological outcomes. The kynurenine pathway is another relevant metabolic branch, linking tryptophan catabolism with immune activity in AKI and CKD (136).

### 6.3 Single-cell transcriptomics and inter-organ immune communication

Cell-resolved studies make the injury states more specific. Ide et al. combined single-cell profiling and experimental perturbation to identify damage-associated proximal tubular cells expressing Sox9, Vcam1, and Cdh6, with reduced homeostatic transport and metabolic genes. These states persisted after severe ischemic injury and were sustained by ferroptotic stress (69). They provide a renal source of prolonged inflammation, but the study did not measure a downstream cerebral response.

On the brain side, the CKD study by Zimmermann et al. combined single-nucleus RNA sequencing, potassium measurements, and functional interventions to connect microglial activation with altered neuronal potassium handling (53). The convergence of cell-resolved analysis and perturbation is more informative than a ligand-receptor prediction alone.

In a 5/6-nephrectomy rat model, hippocampal single-cell analysis identified enriched communication involving C-C motif chemokine ligand 4 (Ccl4) and C-C chemokine receptor 5 (Ccr5). Ccr5 was concentrated in microglia, whereas Ccl4 was expressed by neurons and microglia. Ccr5 knockdown reduced excitatory synapse engulfment and improved synaptic plasticity and memory (137). This connects a cell-resolved signaling hypothesis with functional evidence in a renal-injury model.

Deng et al. analyzed normal mouse pituitary and kidney single-cell datasets, not tissues collected after organ injury. Their computational analysis predicted GH-GHR interactions involving proximal tubular cells and PTN-SDC2/SDC4 and DLK1-NOTCH3 signaling involving renal stromal and vascular populations (138). This provides neuroendocrine communication hypotheses, rather than evidence of an injury-induced kidney-brain signaling event.

Developmental kidney data illustrate another use of the same tools. Matsui et al. combined lineage inference and ligand-receptor analysis in embryonic mouse kidney and tested a predicted nerve-growth-factor effect in organ culture (139). This supports the analytical approach, while remaining distinct from an adult injury atlas or proof of inter-organ signaling.

Ligand-receptor co-expression identifies compatible signaling machinery; it does not demonstrate secretion, transport between organs, or functional receptor activation. Directionality requires paired sampling, spatial localization, circulating mediator measurements, and organ- or cell-specific perturbation. Normal-tissue atlases, kidney-injury datasets, and CKD brain datasets should be kept distinct when constructing an inter-organ model (53, 69, 138).

### 6.4 Multi-omics integration and causal validation

Integrated transcriptomic, proteomic, metabolomic, and clinical data can identify candidate molecular subtypes beyond conventional renal or neurological diagnoses (130, 131). Network analyses can prioritize pathways and compounds, as illustrated by a kidney-Alzheimer disease biomarker analysis (140). Such results need independent experimental replication before they inform treatment selection. A separate machine-learning analysis identified candidate shared biomarkers in diabetic kidney disease and cognitive dysfunction datasets (141), extending the computational landscape without establishing a causal communication pathway.

A translational study should combine serial renal markers, inflammatory proteins, clearance-aware neurological biomarkers, metabolite panels, imaging, and standardized cognitive or functional outcomes. Sampling before and after the relevant insult establishes temporal order. External cohorts and incremental-value analyses then determine whether the integrated signature adds information to routine clinical assessment (130, 131).

## 7 Translational and therapeutic implications

### 7.1 Established clinical management

Management starts with reversible contributors. Uremic encephalopathy is an indication for renal replacement therapy after assessment for competing causes (142, 143). Continuous modalities may be preferable in unstable patients or selected patients with acute brain injury because they produce slower solute and volume shifts (19, 144). Medication review, infection treatment, and correction of glucose, sodium, acid-base, and other metabolic disturbances remain central.

For CKD, care combines vascular risk reduction, appropriate dialysis management, medication review, and cognitive assessment (9, 145, 146). Anemia treatment should follow renal guidance rather than be presented as cognitive therapy: the CHOIR randomized trial found greater risk with a higher hemoglobin target using epoetin alfa (147, 148). Kidney transplantation has been associated with improvements in cognitive and neuroimaging measures, although the observational design does not establish complete reversibility (149).

Conventional dialysis adequacy is also not a sufficient surrogate for cognition. In a maintenance-hemodialysis cohort, lower single-pool Kt/V was not associated with worse cognitive performance (150). Neurological assessment should therefore accompany, rather than be replaced by, solute-clearance indices.

After acute brain injury, kidney protection requires individualized perfusion and volume management, serial urine output and creatinine measurements, sepsis treatment, and review of nephrotoxic medications (2, 151, 152). Low-dose dopamine should not be used to prevent or treat AKI. Diuretics are reserved for appropriate volume management rather than treatment of intrinsic renal injury. Renally eliminated drugs require dose adjustment and therapeutic monitoring when available (151).

Contrast decisions should balance diagnostic benefit against individual renal risk rather than delay necessary imaging on the basis of a blanket assumption of nephrotoxicity (128, 153). Mannitol and hypertonic saline require agent-specific monitoring of fluid balance, kidney function, sodium, chloride, and osmotic exposure (117). Renal replacement prescriptions should account for cerebral hemodynamics and the risk of rapid osmotic shifts (2, 19, 144).

Cognitive problems also affect adherence, communication, and daily function. Coordinated nephrology, neurology, pharmacy, and rehabilitation input can address medication burden and practical care needs (145, 146, 154, 155). Exercise, rehabilitation, and nutritional interventions should be individualized; the evidence for improving cognitive performance in CKD remains heterogeneous (156).

Dietary care should account for CKD stage, nutritional status, electrolyte balance, and the practical effects of cognitive impairment (157, 158). Dietary and rehabilitation measures remain part of individualized care, rather than established treatments for a specific molecular kidney-brain pathway.

### 7.2 Experimental therapeutic strategies

In CKD mice, TRAM34-mediated KCa3.1 inhibition and IL-1 receptor antagonism improved cognitive outcomes (53). Cathepsin C-caspase-8-dependent IL-1β maturation identifies another microglial target (56), while Ccr5 knockdown improved synaptic and memory outcomes in CKD rats (137). NLRP3-GSDMD and neutrophil recruitment provide additional targets from peripheral-inflammation models (57). These results motivate separate pharmacological studies of cytokine processing, neuronal receptor responses, and synaptic engulfment; broader CKD inflammasome mechanisms provide additional targets for experimental evaluation (159). Table 2 distinguishes the models and outcomes supporting these leads.

**Table 2:** Candidate therapeutic strategies for kidney–brain crosstalk.

Strategy	Target or rationale	Evidence studied	Next translational requirement
Microglial signaling	KCa3.1; IL-1 maturation/receptors; Ccl4-Ccr5	KCa3.1/IL-1 interventions improved cognition in CKD mice (53); microglial IL-1β maturation was defined mechanistically (56); Ccr5 knockdown improved cognition in CKD rats (137).	Cell- and pathway-specific target engagement; exposure, host-defense safety, and cognitive outcomes in clinical CKD.
Inflammasome and neutrophil modulation	NLRP3-GSDMD; chemokines; BBB injury	Genetic and inhibitor evidence in peripheral-inflammation mice (57).	Test a causal renal-injury-to-brain pathway before clinical translation.
Ferroptosis and redox interventions	Iron-dependent lipid peroxidation and oxidative injury	Renal AKI and cerebral stroke models, studied separately (71, 165, 166).	Pharmacokinetics in renal dysfunction; tissue selectivity and neurological benefit.
Toxin and microbiota interventions	Retained metabolites and inflammatory burden	Experimental AST-120 cognition data; clinical synbiotic/curcumin surrogate outcomes (169–171).	Neurological endpoints with diet, dialysis, and clearance accounted for.
Stromal-cell-derived EVs	Immunomodulatory and reparative cargo	Experimental CKD literature (175).	Standardize source, cargo, dose, delivery, manufacturing, and safety.
Drug repurposing	Shared disease-associated molecular signatures	Computational prioritization and PC12 cell testing of gefitinib (140).	Validate the target and dose-exposure response in appropriate disease models.

AST-120, oral carbon adsorbent; BBB, blood-brain barrier; CKD, chronic kidney disease; EGFR, epidermal growth factor receptor; EVs, extracellular vesicles; GSDMD, gasdermin D; IL-1, interleukin-1; NLRP3, NLR family pyrin domain containing 3.

Additional immune interventions come from other disease settings. A randomized phase-2 trial of tocilizumab in non-ST-elevation myocardial infarction measured inflammatory and myocardial-injury outcomes (160). B-cell depletion improved pathological and behavioral outcomes in transgenic Alzheimer disease mouse models (161). Neither study established neurological efficacy in patients with kidney disease, so they provide adjacent immunopharmacological evidence rather than kidney-brain treatment recommendations.

Several interventions have been tested directly in CKD-associated neurological models. Tangeretin improved renal and cognitive measures in 5/6-nephrectomized rats (162). Irisin reduced cognitive impairment and hippocampal AhR/NF-*κ*B/NLRP3-related injury in adenine-induced CKD mice (163). Separate muscle-kidney experiments linked circulating irisin to improved tubular metabolism and reduced fibrosis (164). The convergence is biologically informative, but the cerebral and renal mechanisms were not established in one coordinated human intervention.

Ferroptosis inhibitors protect renal tissue in experimental AKI and reduce cerebral injury in stroke models (71, 165). These are separate models, so a benefit in both organs does not demonstrate interruption of kidney-brain signaling. Hydrogen sulfide donors and carnosine also have experimental antioxidant and anti-inflammatory effects (166, 167). Translation requires exposure and safety studies in renal dysfunction, followed by testing of neurological as well as renal outcomes.

Ferrostatin-1 also improved neurological and barrier outcomes in a rat spinal-cord ischemia-reperfusion model through an ERK1/2/SP1/GPX4-associated pathway (168). This is spinal rather than cerebral or renal evidence and should be evaluated accordingly when selecting a translational model.

Microbiota and toxin-directed interventions address a different part of the pathway. AST-120 improved toxin-associated cognitive deficits in experimental models (169). Clinical studies of synbiotics or curcumin in CKD have measured renal, inflammatory, oxidative, or microbial outcomes rather than establishing neurological efficacy (170–173). Future trials should prespecify cognition or neurological function and control diet, antibiotics, dialysis, and residual kidney function.

Portable and wearable artificial kidneys aim to improve continuity of solute and fluid control; neurological benefits remain an outcome to test (174). Mesenchymal stromal-cell-derived extracellular vesicles offer experimental immunomodulatory and reparative mechanisms in kidney disease. Their development requires standardized source, cargo, dose, manufacturing, and safety assessment before a kidney-brain indication can be evaluated (175).

Drug repurposing can combine computational prioritization with early experimental testing. Cai et al. identified EGFR-related signatures and included gefitinib in experiments using A*β*-injured PC12 cells (140). This is preclinical evidence from a cell model, rather than an established intervention for renal-disease-related brain injury. Target engagement, organ-specific effects, and dose-exposure relationships need testing in models that connect renal injury to neurological outcomes before clinical evaluation.

A different repurposing example used metformin pretreatment in nondiabetic female mice with CKD before experimental ischemic stroke. Treatment reduced infarct injury and improved neurological outcomes alongside AMPK-related changes (176). This model provides a direct renal-disease-plus-brain-insult experiment, but does not establish the benefit or dosing of metformin for neurological prevention in people with advanced CKD.

## 8 Discussion and future research

Kidney-brain crosstalk is supported by clinical associations and increasingly specific experimental mechanisms, but the evidence does not form one uninterrupted causal chain. Kidney function influences blood neurological biomarkers (26, 27); systemic illness and treatment influence AKI after brain injury (2). The key task is to determine which pathways account for outcome differences after these influences are considered.

Longitudinal studies should distinguish brain-driven signals, secondary insults, and shared baseline vulnerability. Serial measurements can establish whether a molecular change precedes renal or neurological deterioration. Kidney and brain tissue studies should use cell-specific perturbations to test proposed links, including microglial cytokine processing and synaptic engulfment, GSDMD signaling, ferroptotic stress, endocrine changes, and impaired cerebral clearance. Integrating these experiments with clinical omics requires standardized outcomes and independent validation (130, 131).

Intervention studies should state whether they seek renal protection, neurological protection, or modification of a shared inflammatory process. Cognition, delirium, neurological recovery, kidney recovery, and safety should be assessed alongside target engagement. Endocrine and glymphatic observations are priorities for mechanistic work, not yet established treatment targets. Biomarker-based enrichment becomes useful when it adds information beyond kidney function, illness severity, and existing clinical predictors.

## 9 Conclusions

AKI, CKD, and acute brain injury are connected by shared vascular vulnerability and bidirectional metabolic, immune, autonomic, and endocrine responses. Experimental studies identify specific endothelial, microglial, neuronal, and tubular mechanisms, while Klotho-FGF23 changes and perivascular clearance findings extend the model of chronic brain vulnerability. Clinical care centers on reversible metabolic disturbances, appropriate perfusion and fluid management, and medication-related risk. Further progress depends on linking cell-specific mechanisms to longitudinal human outcomes and testing whether targeted interventions improve both organ function and patients' neurological recovery.
